# Correction: Effect of molybdenum carbide concentration on the Ni/ZrO_2_ catalysts for steam-CO_2_ bi-reforming of methane

**DOI:** 10.1039/d0ra90001j

**Published:** 2020-01-22

**Authors:** Weizuo Li, Zhongkui Zhao, Panpan Ren, Guiru Wang

**Affiliations:** State Key Laboratory of Fine Chemicals, Department of Catalysis Chemistry and Engineering, School of Chemical Engineering, Dalian University of Technology Dalian 116024 P. R. China zkzhao@dlut.edu.cn +86-411-84986354

## Abstract

Correction for ‘Effect of molybdenum carbide concentration on the Ni/ZrO_2_ catalysts for steam-CO_2_ bi-reforming of methane’ by Weizuo Li *et al.*, *RSC Adv.*, 2015, **5**, 100865–100872.

The authors regret that incorrect TEM images were shown in Fig. 7 of the original article. The authors would like to use the following correct TEM images, shown in Fig. 7 below. The authors state that this error has no effect upon the conclusions of the article.

**Fig. 7:**
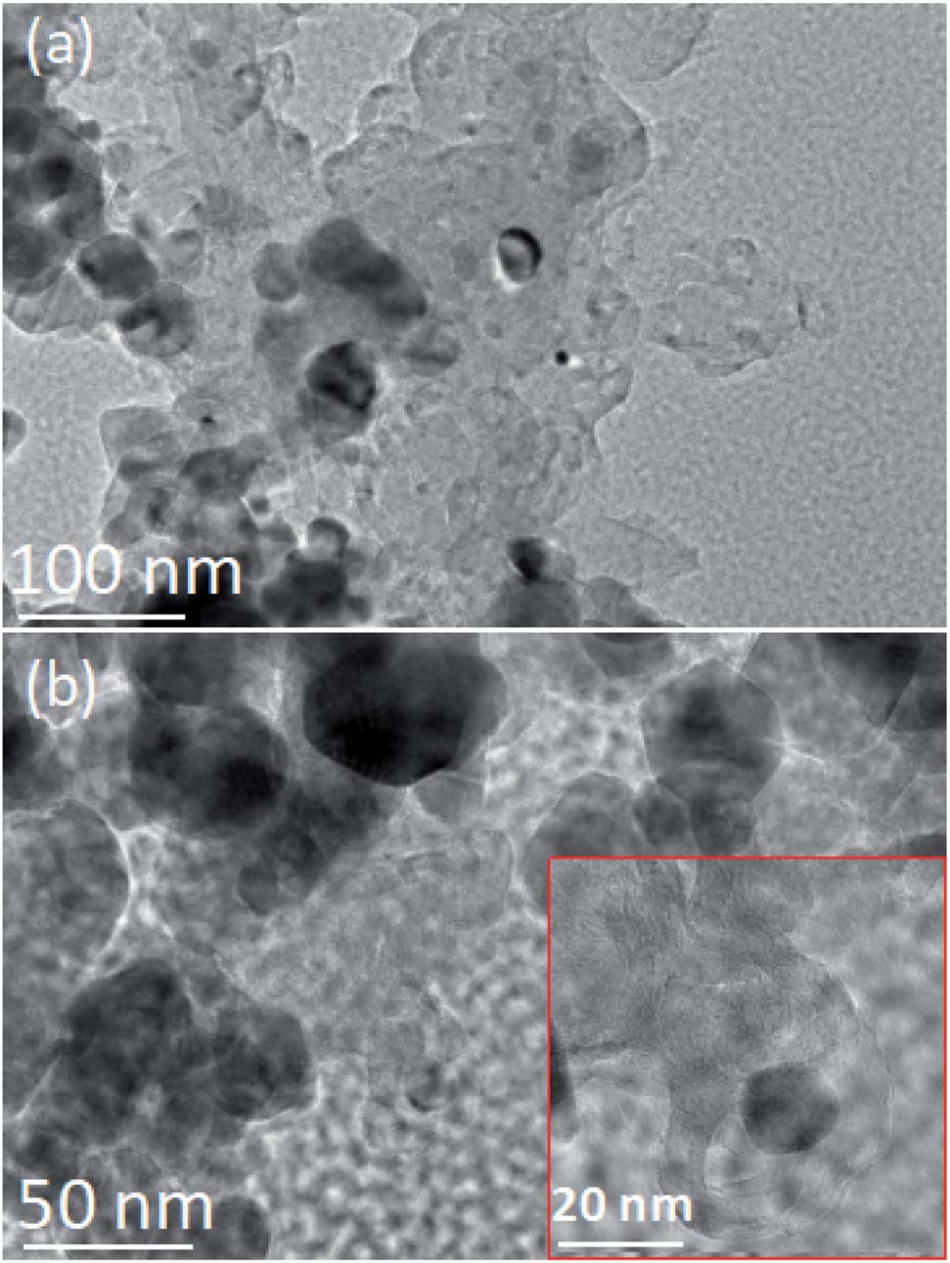
TEM images of coke on the spent Mo_2_C–Ni/ZrO_2_ (a) and Ni/ZrO_2_ (b) catalysts. Inset in (b) is magnified shell-like carbon.

The Royal Society of Chemistry apologises for these errors and any consequent inconvenience to authors and readers.

## Supplementary Material

